# Exploring Heterogeneity in perinatal depression: a comprehensive review

**DOI:** 10.1186/s12888-023-05121-z

**Published:** 2023-09-04

**Authors:** Ahmed Waqas, Mahnoor Nadeem, Atif Rahman

**Affiliations:** 1 Department of Primary Care & Mental Health, Institute of Population Health, University of Liverpool, Liverpool, UK; 2 Sabancı University, Istanbul, Turkey

**Keywords:** Perinatal depression, Trajectories, Heterogeneity, Postnatal depression, Postpartum depression, Phenotypes

## Abstract

Perinatal depression (PND) is a significant contributor to maternal morbidity globally. Recognized as a major cause of poor infant development, epidemiological and interventional research on it has increased over the last decade. Recently, studies have pointed out that PND is a heterogeneous condition, with variability in its phenotypes, rather than a homogenous latent entity and a concrete diagnosis, as previously conceptualized in psychometric literature and diagnostic systems. Therefore, it is pertinent that researchers recognize this to progress in elucidating its aetiology and developing efficacious interventions.

This systematic review is conducted in accordance with the Meta-analysis of observational studies in epidemiology (MOOSE). It aims to provide an updated and comprehensive account of research on heterogeneity in phenotypes of PND and its implications in research, public health, and clinical practice. It provides a synthesis and quality assessment of studies reporting heterogeneity in PND using cutting-edge statistical techniques and machine learning algorithms. After reporting the phenotypes of PND, based on heterogeneous trajectories and symptom profiles, it also elucidates the risk factors associated with severe forms of PND, followed by robust evidence for adverse child outcomes. Furthermore, recommendations are made to improve public health and clinical practice in screening, diagnosis, and treatment of PND.

**Supplementary Information:**

The online version contains supplementary material available at 10.1186/s12888-023-05121-z.

## Introduction

Perinatal depression (PND) is prevalent and significantly contributes to maternal and infant morbidity globally [1–3]. Globally the prevalence of depressive symptoms during the antenatal period is estimated at 28.5% and 27.6% during the postnatal period [4]. The onset of PND is largely attributed to socio-environmental, cultural, and financial stressors [3, 4]. Furthermore, hormonal changes experienced during pregnancy, such as changes in progesterone and glucocorticoids, increase women’s susceptibility to depression during the perinatal period [5–7]. Recent reviews have revealed that PND is associated with poor child outcomes [3], including low birth weight infants, preterm deliveries, underweight and stunting, poorer breastfeeding practices, higher incidence of infectious illnesses, and poorer emotional, motor, and language development. Thus, PND poses a significant threat to maternal and child health globally.

PND is a well-known contributor to maternal and infant morbidity and accounts for major economic losses [8]. However, it has gained little attention from the research community dedicated to studying major depressive disorders in other populations. This lack of research has impeded our understanding of the nature of PND, which is required to develop more effective treatments [9]. One challenge in understanding PND is its heterogenous clinical presentation [10]. These heterogeneous presentations of PND or its endophenotypes represent distinct constellations of symptoms that may have specific etiological and genetic underpinnings. Galea and Frokjaer [10] have emphasized that elucidation of phenotypic heterogeneity in PND is necessary to develop more personalized treatment approaches and more effective pharmacological treatments. This heterogeneity in PND may be due to heterogeneous symptom constellations or indicators of longitudinal trajectories such as time of onset, chronicity, recurrence, and hormonal and genetic markers [10]. For instance, using data-driven approaches, Waqas and Rahman showed that Pakistani women with perinatal depression present with four distinct phenotypes: mild depression, mixed anxiety-depression, somatic depression, and atypical depression [11]. Moreover, these subtypes of PND are associated with variable prognoses [12].

Santos Jr. et al. provide an excellent review of studies on heterogeneous presentations of PND [13] with varying longitudinal trajectories and symptom profiles. While presenting this evidence, they argue that our understanding of PND is still lacking primarily due to conceptualizing PND as a unidimensional, homogenous, and concrete latent construct underpinned in an essentialist paradigm. These assumptions are problematic and have slowed down depression research [14, 15]. Furthermore, these assumptions have essentially undermined the complexity of PND, which further translate to a lack of personalized and effective preventive and treatment approaches.

This systematic review aims to provide an up-to-date synthesis of research on heterogeneity in perinatal depression. It builds on previous reviews by Santos Jr. et al. and Baron et al. who presented literature on heterogeneity in perinatal depression using latent class, growth mixture, and trajectory analysis approach. This review aims to synthesize findings and assess the quality of literature presenting heterogenous symptomatic presentations of PND and its longitudinal trajectories. It also aims to synthesize evidence on their risk factors, prognosis with different treatment approaches, and association with maternal and child outcomes.

## Methods

### Database search

This systematic review is conducted per the Preferred Reporting Items for Systematic Reviews and Meta-Analyses (PRISMA) and the Meta-analysis of observational studies in epidemiology (MOOSE) guidelines for systematic reviews [16, 17]. The protocol for this systematic review was registered a priori in PROSPERO [18]. We searched PubMed and Web of Science, through 21^st^ February 2022, using a pretested search strategy encompassing terms about heterogeneity and perinatal depression (Table 1).

**Table 1:** Search strategy adapted for PubMed

Concept	Keywords
**Perinatal depression**	("postnatal depression"[Title/Abstract] OR "depression, postpartum"[MeSH] OR "antenatal depression"[Title/Abstract] OR "antepartum depression"[Title/Abstract] OR "prenatal depression"[Title/Abstract] OR "maternal depression"[Title/Abstract] OR "perinatal depression"[Title/Abstract] OR "peripartum depression"[Title/Abstract] OR "postpartum depression"[Title/Abstract])
**Heterogeneity**	(heterogen*[Title/Abstract] OR trajector*[Title/Abstract] OR network*[Title/Abstract] OR "growth curve*"[Title/Abstract] OR "mixture model*"[Title/Abstract] OR subtyp*[Title/Abstract] OR phenotyp*[Title/Abstract] OR latent-class*[Title/Abstract] OR latent-profil*[Title/Abstract] OR cluster-analys*[Title/Abstract])

### Inclusion & exclusion criteria

- We considered all studies that assessed depressive symptoms from pregnancy to 1 year postpartum, using psychometric rating scales or diagnostic criteria (DSM or ICD).
- We included all studies which reported heterogeneity in perinatal depression among pregnant or postpartum women aged 18 years or older, irrespective of their study designs.
- Studies reporting different symptom profiles of PND and longitudinal trajectories such as stability of symptom severity, chronicity, persistence, and remission were considered.
- We also included studies that explored the effectiveness of different treatments and prognoses of PND in heterogenous subtypes of PND.
- We also considered studies which reported associations of heterogeneous PND presentations with child health outcomes.
- Only original articles describing heterogeneity in perinatal depression using data-driven approaches (such as cluster analysis or principal component analysis) or statistical and epidemiological approaches (including mixture modelling, latent class analysis, or growth curve modelling) were considered.
- We considered those studies with ≥ 500 study participants, to ensure studies were adequately powered. This was important because machine learning and statistical approaches for assessing heterogeneity require higher sample sizes.
- We excluded short formats of publications such as letter to editors and correspondence.

### Operational definitions

#### Prognostic dichotomy

For this review, we classed different heterogeneous classes or trajectories as either complex or standard. This dichotomy was based on prognosis of each class or trajectory. Classes or trajectories associated with worse prognosis or outcomes were classed as “complex” or termed as ‘a severe and debilitating’. This dichotomy was applied uniformly to heterogenous classes of PND based on either symptom profile, time of onset or trajectories.

#### Heterogeneity

Heterogeneity in major depressive disorders has attracted significant attention. There has been some interest on heterogeneity in PND as well, especially after the landmark studies from the *Postpartum Depression: Action Towards Causes and Treatment (PACT) Consortium* are worth mentioning here [19, 20]. According to the PACT consortium [19, 20] and Baron et al. [21], there are three sources of heterogeneity in PND, which were considered for review:

1. Symptom profiles where perinatal women could be divided into distinct symptom clusters or clinical phenotypes [19, 20].
2. Temporal onset where the debate on the time of onset of PND symptoms has attracted major attention. According to Putnam and colleagues [19, 20], this debate about the temporal onset and duration of PND has important implications for the field. The temporal onset of PND (antenatal or postnatal; early or late) maybe the key to disentangle the pathophysiological processes underlying this condition.
3. Temporal trajectories [21] where different perinatal women present with different patterns of chronicity and relapse.

### Data extraction procedures

After searching academic databases, duplicate entries were removed using Endnote v. X9. After that, eligible studies were filtered in a two-phased screening process by two independent reviewers. First, the reviewers extracted study design and publication characteristics such as the scope of the study, country, setting, and sampling technique. Characteristics related to the study sample included inclusion and exclusion criteria, timepoints for data collection, psychometric tools, and diagnostic criteria used to assess PND. After that, statistical techniques used to analyze heterogeneous profiles of PND were enumerated. Findings were synthesized narratively according to their themes, such as the description of the study's heterogenous symptom profiles or trajectories, risk factors, child outcomes, and treatment considerations.

### Quality assessment

Quality assessment was done using an adapted version of The Newcastle–Ottawa Scale (NOS) for assessing the quality of nonrandomised studies in meta-analyses [22]. All studies were scored for quality across five domains, including appropriateness of study sample, comparability of the cohort based on design and analysis, adequacy of statistical analysis, attrition, and adequacy of outcome assessment. Each domain was rated as adequate, partly adequate, and inadequate.

We assessed the adequacy of the study sample by evaluating its representativeness of the population under study in each individual research. This evaluation was based on several factors, including the inclusion and exclusion criteria used in the studies and the reported characteristics of the participants. Specifically, we examined the recruitment methods used in each study and compared the demographic and clinical characteristics of the study participants with those of the broader population of interest.

The evaluation of exposure was deemed as either adequate or partially adequate based on the methods used for participant recruitment. Specifically, if diagnostic criteria were applied, the ascertainment of exposure was considered adequate. In contrast, if psychometric scales were used, it was rated as partially adequate. As for the assessment of outcome, it was deemed adequate if validated scales were employed to measure depressive symptoms, thereby facilitating the elucidation of symptom profiles or trajectories. Attrition rate was rated adequate if it was ≤ 20%. Statistical analysis was judged as adequate if established statistical methods were used for modelling heterogeneity in PND followed by use of established statistical criteria for retaining number of classes/subgroups.

### Quality of reporting in studies reporting longitudinal trajectories

We also utilized the Guidelines for Reporting on Latent Trajectory Studies (GRoLTS) to assess the quality of reporting in the studies reviewed [23]. The GRoLTS checklist standardizes the reporting of results from latent trajectory analyses. This checklist was particularly relevant for our review, given its focus on studies employing latent growth mixture modeling (LGMM) or latent class growth analysis (LCGA). We systematically evaluated each study against the GRoLTS key components, identifying potential weaknesses and gaps in reporting. This checklist includes 16 key components that are crucial for reporting results of trajectory studies. These components cover a range of aspects, including the metric of time used in the statistical model, handling of missing data, distribution of observed variables, software used, consideration of alternative specifications of within-class heterogeneity and between-class differences, use of covariates, number of random start values and final iterations, model comparison tools, total number of fitted models, number of cases per class, entropy, plots of estimated mean trajectories, characteristics of the final class solution, and availability of syntax files [23].

### Data analysis

A narrative synthesis approach was used to synthesize evidence in this systematic review. Meta-analyses were not conducted due to heterogeneity in study designs and outcomes included in the review.

## Results

**Fig. 1:**
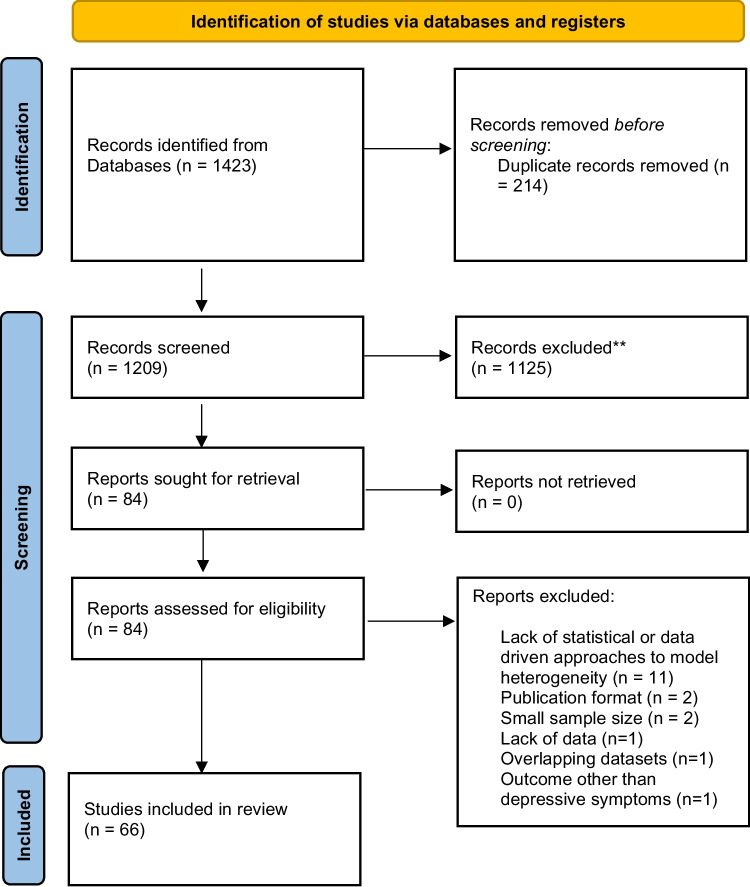
PRISMA flowchart presenting selection of studies included in the review

The database search yielded a total of 1423 bibliographic records. After deleting duplicate records, 1209 titles and abstracts were screened during the first phase. Of these, 1125 did not fulfil all the eligibility criteria; therefore, 84 studies were included in the full text review. In this phase, major reasons for exclusion of articles were lack of statistical or data-driven approaches for modelling heterogeneity (*n* = 11), outcome other than depressive symptoms (*n* = 1), publication format (*n* = 2), small sample size (*n* = 2), lack of data (*n* = 1) and overlapping datasets (*n* = 1). 66 full texts were considered eligible for data extraction (Fig. 1).

Most of the research evidence came from high income countries (*n* = 49, 74.24%), followed by upper-middle-income countries (*n* = 11, 16.67%), low middle income countries (*n* = 3, 4.55%). Three of the studies utilized data from multiple countries (*n* = 3, 4.55%). USA and Australia contributed the greatest number of studies (*n* = 23, 34.84%). Most of the studies were based on secondary statistical analyses of datasets available (*n* = 46, 69.70%). The sample size of the studies ranged from 501 to 17,912 participants.

Most of the studies reported longitudinal heterogeneity accounting for severity and chronicity (*n* = 53), heterogeneous subtypes based on biopsychosocial factors (*n* = 7), heterogeneous subtypes based on symptoms of depression (*n* = 6), and heterogeneity based on the period of onset (*n* = 5). Child outcomes were reported in 26) studies, adverse pregnancy outcomes (*n* = 2), prognosis after delivery of the intervention (*n* = 2), and symptom networks (*n* = 2).

### Quality assessment

**Fig. 2:**
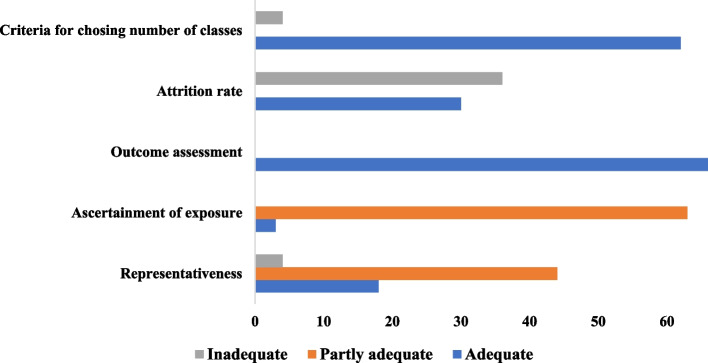
Quality assessment of studies

Generally, the quality of evidence presented by these studies was robust. Representativeness of the study sample was judged as adequate in 18 (40.9%) of the studies and partly adequate in 66.67% of the studies. Ascertainment of exposure was done using clinical diagnoses in only three studies (4.55%) and psychometric scales among the rest. Outcome assessment was done using psychometrically sound scales in all the studies. The extent of missingness and attrition was > 20% among 36 (54.55%) studies, while all but four studies (6%) used appropriate statistical analysis techniques and appropriate criteria for choosing the number of classes/trajectories of PND in their study samples (Fig. 2).

**Fig. 3:**
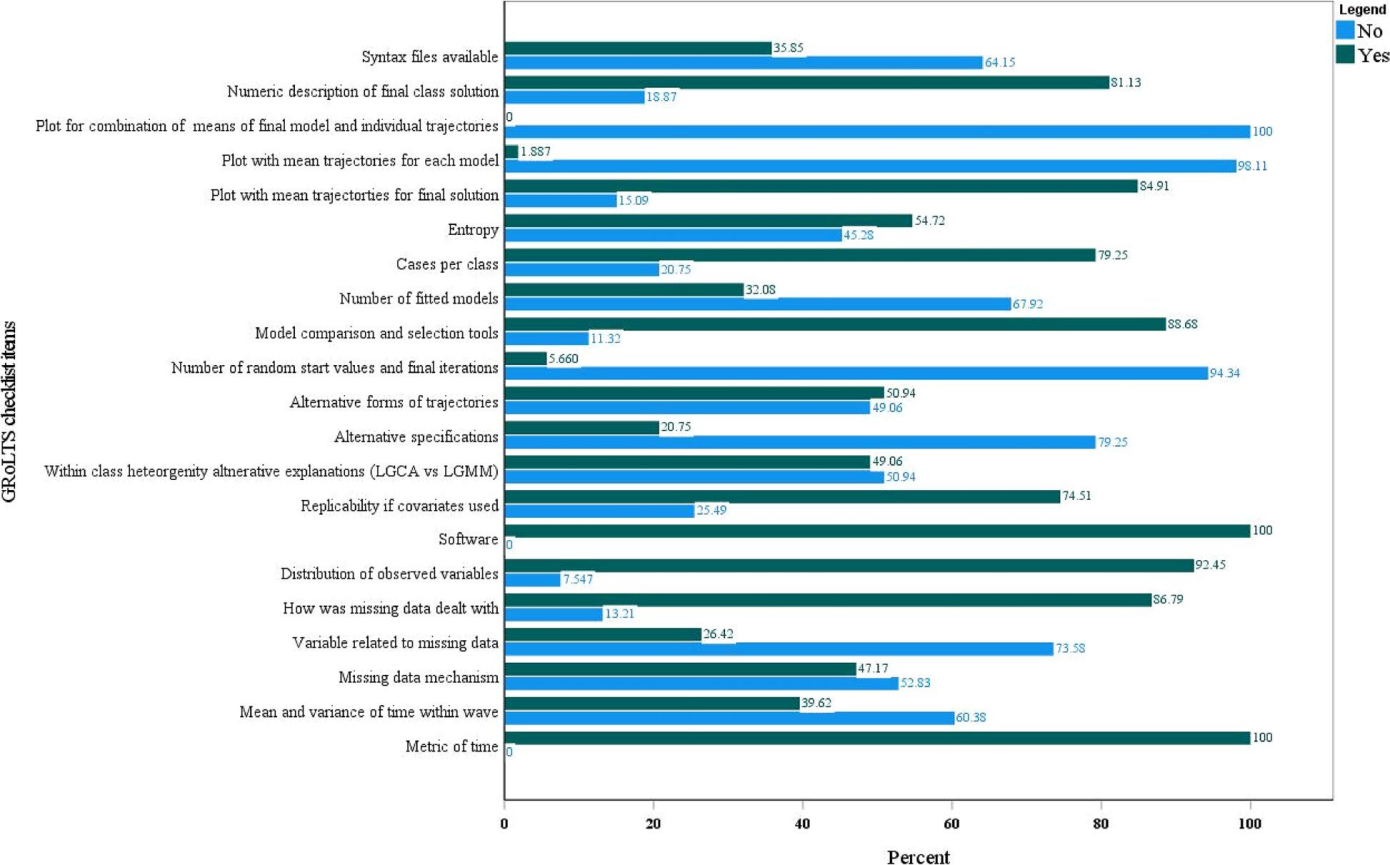
Quality of reporting in studies on longitudinal trajectories of perinatal depression as per the GRoLTS checklist

We assessed the quality of reporting in the included studies (*n* = 53) using the GRoLTS checklist (Fig. 3, Supplementary file 2). Our findings indicate that all studies reported the metric of time used in the statistical model and the software used. The majority of studies also provided information on how missing data were dealt with (86.8%), the distribution of observed variables (92.5%), and included a plot with the mean trajectories for the final solution (84.9%). Furthermore, 81.1% of studies provided a numeric description of the final class solution, and 79.2% reported the number of cases per class.

However, there were several areas where reporting could be improved. Only 39.6% of studies presented information about the mean and variance of time within a wave, and less than half (47.2%) reported the missing data mechanism. Only 26.4% of studies provided a description of what variables were related to missing data. While 74.5% of studies ensured replicability if covariates were used, only 49.1% considered alternative explanations for within-class heterogeneity (LGCA vs LGMM), and 50.9% described alternative forms of trajectories.

Furthermore, only 32.1% of studies reported the total number of fitted models, and a mere 5.7% provided information about the number of random start values and final iterations. Only 54.7% of studies reported entropy, and just 1.9% included plots with the mean trajectories for each model. None of the studies included a plot of the combination of estimated means of the final model and the observed individual trajectories. Lastly, only 35.8% of studies made their syntax files available.

### Heterogeneous symptoms profiles of PND

#### General characteristics

Different analytical techniques underpin the heterogeneous profiles of PND in this body of literature (*n* = 13) (Supplementary Table 1). These generally reported large sample sizes ranging from 515 to 17,912, with only two studies in low- and middle-income countries [24, 25]. Seven of these studies recruited women during the antenatal period, four during the perinatal period and two during postpartum. These studies utilized latent class analysis (*n* = 4), cluster analysis (*n* = 2), factor analysis (*n* = 2), and network analysis (*n* = 2) and regression analysis (*n* = 3).

#### Consideration of onset period

Three of these [26–28], considered the period of onset of PND symptoms. Aoyagi et al. [26] and Tebeka et al. [28] presented three subgroups of postpartum women: no postpartum depression, early onset, and late-onset postpartum depression. Less than 1/4^th^ of the study samples in their respective studies sample were screened for early or late onset PND. In comparison, Fransson et al. [27] yielded four subtypes: no symptoms, antenatal (8.4%), postpartum (11.23%), and persistent depression (16.03%).

#### Psychosocial stratification

Eastwood et al. considered Psychosocial stratification of antenatal indicators to guide population-based programs in perinatal depression [29]. Using Latent Class Analysis (LCA), they found four distinct patient groups based on their migration status and presence of distress.

#### Clinical phenotypes based on symptoms

Six studies [24, 25, 30–33] reported clinical phenotypes of PND based on symptoms of perinatal depression, utilizing a mixture of techniques: LCA, k-means cluster analysis, two-step cluster analysis, factor analysis, and logistic regression and mixed model analyses. These studies presented three to five distinct subtypes of perinatal depression. Putnam et al. [30, 31] utilized LCA to delineate heterogeneity in postpartum depression by utilizing data records of 17,912 postpartum women in seven countries. They reported that among all the women, irrespective of caseness, there were three distinct latent classes with the most striking characteristics associated with severity, the timing of onset, comorbid anxiety, and suicidal ideation. The most severe symptoms of postpartum depression were significantly associated with poor mood, increased anxiety, the onset of symptoms during pregnancy, obstetric complications, and suicidal ideation. While in class 2 (moderate severity), most women reported symptom onset within four weeks postpartum and had more pregnancy complications.

The same dataset was utilized to identify symptom dimensions of postpartum depression among 663 women with perinatal depression [31]. It utilized a two-tiered analytical approach of principal component analyses and cluster analyses, yielding three symptom dimensions on EPDS: depressed mood, anxiety, and anhedonia. K-means cluster analyses identified five subtypes of women with PND: severe anxious depression, moderate anxious depression, anxious anhedonia, pure anhedonia, and resolved depression [31]. Thoughts of self-injury were more frequent among subtypes with comorbid anxiety and, to a lesser extent, in pure anhedonia.

Three studies [24, 25, 32] reported largely overlapping subtypes of PND. Both these studies identified subgroups of women with somatic symptoms and mild depression. Sun et al. utilized the PHQ-9 scale to delineate these subgroups; therefore, they could not account for anxiety symptoms [24]. While Saldana et al. and Waqas & Rahman, utilizing more elaborate scales (Beck Depression Inventory, Postpartum Depression Screening Scale, and Hamilton Depression Rating Scale) in their studies, identified distinct subtypes demonstrating comorbid anxiety and cognitive symptoms. Both these studies also identified a symptom dimension exhibiting insomnia symptoms [25, 32].

#### Network analytical approaches

Two studies utilized a novel analytical approach of network analyses with the Gaussian Graphical Model approach, utilizing symptom level data on psychometric scales [34, 35]. Phua et al. [34] reported several essential insights pertaining to symptom networks utilizing symptom level data on EPDS and State-Trait Anxiety Inventory among perinatal women in Singapore. They reported that among perinatal women, connections among antenatal and postnatal depressive symptoms become more substantial over time as they keep reinforcing each other. Therefore, persistent depressive symptoms have a poorer prognosis. Significant qualitative differences exist in depressive symptoms across the perinatal period. Cognitive-affective symptoms are more central during pregnancy, including feelings related to low self-esteem, worrying thoughts and agonizing over past failures. During the postpartum period, the sense of being overwhelmed or being punished was most central in the depressive-anxiety network. When overlaps between symptoms of anxiety and depression were considered, feelings of guilt, nervousness, or being a failure during pregnancy acted as bridging symptoms. After pregnancy, the top bridging symptoms were related to self-blame, feeling overwhelmed, and excessive worries [34].

#### Quality assessment

The studies included in this section were generally robust. The sample size in each study was adequate, ranging from 515 to 17,912. However, five of the studies reported either non-response rates or missingness more than 20% [19, 20, 24, 28, 35]. All studies were either fully or partly representative of the population under studies. All studies utilized either valid and reliable diagnostic criteria or psychometric scales for ascertainment of exposure. Moreover, statistical methods and criteria for retaining number of subclasses/model fit statistics were robust. It is also worth noting that only Tebeka et al., used diagnosis of PND among participants to classify heterogeneous profile using logistic regression approach [28].

### The longitudinal trajectory of PND symptoms

A total of 53 studies presented evidence of heterogeneity in longitudinal trajectories of PND. A higher proportion of the studies (14, 26.42%) were conducted during the postpartum period, followed by during pregnancy (38, 71.70%). Only one study recruited study participants during both the pregnancy and postpartum periods. Various statistical techniques were utilized to assess longitudinal heterogeneity in perinatal depression. The most frequently utilized statistical analysis technique was growth mixture modelling (*n* = 13), k-means cluster analysis (*n* = 1), latent class (*n* = 4) and latent class growth analysis (*n* = 6), group-based trajectory modelling (*n* = 4), and semi-parametric mixture modelling (*n* = 12) and latent profile analysis (*n* = 2). Timepoints for data collection ranged from a minimum of two to ten. EPDS (*n* = 31) and CES-D (*n* = 12) were the most frequently used scale in analyses.

In the following sections, we will provide a more nuanced understanding of the interconnected aspects of pattern, severity, chronicity, and onset of symptoms, and their collective influence on the trajectory of Perinatal Depression (PND). Recognizing the complexity of PND, our discussion is structured into two interconnected sub-themes. The first, 'The longitudinal trajectory of PND symptoms: An examination of severity and chronicity' , explores how the intensity and duration of symptoms can shape the course of PND. The second, 'The longitudinal trajectory of PND symptoms: A focus on onset during prenatal and postnatal periods', investigates how the timing of symptom onset, whether during the prenatal or postnatal period, can influence the progression of PND [13, 21].

While this categorization may seem artificial due to the intertwined nature of these aspects, it is guided by the variables used by authors of the primary studies to derive the latent trajectories of PND. This approach underscores the importance researchers place on these aspects when studying longitudinal trajectories of PND. Furthermore, the distinction between trajectories based on severity and chronicity versus onset timing underscores the complexity of PND and suggests that postnatal depression may often be a continuation of antenatal depression. This observation is crucial in understanding the differences between the DSM-V's perinatal specifier and the ICD-10's focus on postpartum depression alone [19, 20]. Through this approach, we aim to offer valuable insights into the heterogeneity of PND and inform more effective strategies for its screening, diagnosis, and treatment.

#### The longitudinal trajectory of PND symptoms: An examination of severity and chronicity

Among the 53 studies, 32 studies (60.38%) reported longitudinal trajectories based either on severity or chronicity (or both) of perinatal depression (Supplementary Table 2). These studies represented a total sample size of 132,899 women, ranging from 501 to 15,590. The number of classes of trajectories ranged from two [36–40] to seven [41]. In this body of literature, PND symptoms were visualized in terms of severity on onset (mild, moderate, and severe), stability (stable, decreasing, and increasing), or trend presenting stable linear growth overtime or unstable trajectories with at least one trajectory depicting a quadratic trend.

Four studies reported two trajectories of PND [36, 38–40]. Three of these studies were conducted during the antenatal period. For the collection of data on PND symptoms, these studies employed three to four assessment waves using psychometric scales. The longest follow-up assessment was conducted at 21 years postpartum [40]. Heterogenous nomenclature was adopted to identify patterns of PND in these studies. The trajectories reported were either stable (persistently low or high) or unstable (escalating, decreasing) [36, 38–40], with most participants reporting persistently low or decreasing trajectories over time.

Seven studies reported three longitudinal trajectories of perinatal depression [42–48]. All studies except one recruited study participants during the antenatal period [42]. Three of these studies presented trajectories based on symptoms scores: low, moderate, and high or improved, stable and worsened [43, 46, 48]. In comparison, the rest of the studies presented trajectories accounting for the level of symptom severity at onset (low, moderate, and high) and the trend (stable, increasing, and decreasing) after that [42, 44, 46, 47]. Among these studies, about 9% of the study participants presented with the worst prognosis.

Eleven studies [49–59] reported four trajectories either based on the trend of depression severity over time, a combination of scores on psychometric scales and time of onset, or the trend after that. All but three studies recruited participants during the antenatal period [49–51]. Waves of data collection ranged from three [59] to eight [60]. The most extended follow-up was conducted by Ferro et al. where women were followed up to 15 years postpartum [49]. Based on the severity of scores at onset, all the studies reported a resilient group of women who reported minimal depressive symptoms throughout the study period [50–59]. In addition to these variables, Ladyman et al. also accounted for the ethnicity of study participants [59]. While comparing ethnic inequities in health between Māori and non-Māori in New Zealand, persistent clinically significant depressive symptoms were found among the former group.

Ten studies reported more than four trajectories [41, 61–68]. All but three studies recruited postpartum women [61, 62, 68], reporting analysis of three [63] to eight [41] data collection waves. Only one study [61] reported five trajectories based on the timing of onset (Never, antepartum only, postpartum, late, and chronic). PND presenting as either during antepartum or postpartum subsided over time. However, late-onset patients reported elevated depressive symptoms during the second year postpartum. Netsi et al. utilized data from the ALSPAC cohort comprising 15,427 women between 2 to 134 months postpartum. They evaluated the persistence of EPDS scores across eight timepoints. Depression was defined as an EPDS score above the threshold level at 2 and 8 months after childbirth, yielding seven unique trajectories: below the threshold, moderate but not persistent, marked but not persistent, severe but not persistent, moderate persistent, marked persistent and severe persistent [41]. The rest of the studies presented a similar trajectory pattern classified as either linear or quadratic based on the severity of PND symptoms. These studies presented that an estimated 2.80% of women suffer from persistently high and chronic depressive trajectories.

All the studies in this section were generally of good quality, with large sample sizes. However, many of these studies (*n* = 16) suffered from high rates of attrition across waves of assessments [40–46, 49, 50, 52, 53, 57, 58, 61, 64, 69]. Eight of the studies were adequately representative of the population [36, 49, 57, 63, 66, 67, 69, 70]. A total of 21 studies were rated as partly representative of the population under study, due to class imbalance or underrepresentation of specific groups of participants [38, 40, 41, 43–46, 50–52, 58–61, 64, 68, 71–75]. All the studies utilized psychometric scales for ascertainment of exposure, and statistical methods.

#### The longitudinal trajectory of PND symptoms: A focus on onset during prenatal and postnatal periods

Based on severity scores and onset timing, 21 (out of 53) studies presented heterogeneous patterns of PND (Supplementary Table 3). The trajectories ranged from two [76] to seven [77, 78]. Their sample sizes ranged from 776 [79] to 7223 [77, 78]. All of these were conducted in high-income countries except for two cohorts in Ghana and Chile [79, 80]. All but one [80] were conducted during the antenatal period. Two cohorts focused on at-risk populations [76–78]. Betts et al. utilized data from Mater University Study of Pregnancy, with the study sample skewed toward lower sociodemographic backgrounds. They were more likely to report a history of smoking and being unmarried during pregnancy than the population average [77, 78]. Glasheen et al. used data from two longitudinal studies recruiting substance users; to study the effects of prenatal alcohol and marijuana exposure on offspring development [76].

The most straightforward trajectory pattern of PND comprised of two heterogenous groups: low prenatal and postnatal depression and high prenatal and postnatal depression, where most participants belonged to the latter group [76]. Three studies [79, 81, 82] reported three trajectory patterns, and six studies reported four to five trajectories [80, 83–87], providing follow-ups ranging from 24 months [83, 84] to 14.6 years [80] postpartum. All these studies used a mixture of linear and quadratic trends across pregnancy and postpartum, with both stable and escalating trajectories. Betts et al. in two studies using the same dataset, reported seven trajectories accounting for symptoms of depression, anxiety, and distress [77, 78].

Two studies reported trajectories quantifying risk; the low-risk sample comprised approximately 90% of the study participants [88, 89]. In addition, other trajectories identified in these two studies were: high-risk (persistent high depressive symptoms but below the cut-off for minor depression [88]; early risk (initially high severity of depression but declining over time) and late risk characterized by increasing depressive symptoms and decreasing parental self-efficacy throughout the study period [89].

A total of seven studies conceptualized depressive symptom trajectories using cut-offs with clinical relevance [37, 90–95]. Five of these studies reported three trajectories, and the rest yielded seven trajectories [94, 95]. Pellowski et al. [94] accounted for both severity of depressive symptoms across the study period and their variation in the antenatal and postnatal periods. The rest of the studies visualized these trajectories based on extent and speed of remission and improvement [90] and cut-off value fulfilling criteria for minimal, subclinical, and clinically high symptom severity [91, 93, 95]. While two studies [37, 92] reported trajectories based on severity and chronicity (decreasing and increasing corresponding to resolution and worsening of symptom severity). About 7.6% of these study samples reported worse clinical prognoses.

Quality assessment revealed these studies to be generally robust. However, 14 of the studies reported inadequate rates of attrition or missingness [37, 76–78, 80, 81, 83, 85, 86, 88, 89, 93–95]. Only four of the studies [37, 80, 84, 91] recruited participants which were representative of the general population. All studies used robust statistical techniques for data analysis.

### Risk factors for PND subtypes with worse clinical prognosis

Risk factors for PND trajectories in 40 of the studies (Table 2). Among these studies, predictors of more debilitating trajectories included psychosocial, biological, and environmental factors. Among psychosocial risk factors, the most frequently reported risk factors included poorer marital relationships, poor social support, unwanted pregnancy, stressful life events, low income and financial difficulties and belonging to ethnic minorities. In addition, the presence of anxiety and stress during pregnancy and history of depression were among significant psychiatric risk factors.

**Table 2:** Risk factors associated with more severe and debilitating trajectories of PND

Domain	Risk factors	Number of studies reporting the risk factor
Psychosocial	Marital relationship [33, 36, 38, 40, 42–46, 48, 53, 62, 65, 68, 77, 95, 96]	16
	Low income and financial problems [37, 43, 45, 46, 48, 53, 60, 62, 65, 76, 79, 85, 96]	13
	Poor social network and support [40, 44–46, 53, 64, 68, 71, 76, 97]	10
	Ethnic minority [36, 47, 56, 57, 61, 65, 83, 91, 97]	9
	Unwanted pregnancy [38, 40, 47, 48, 61, 62, 64]	7
	Stressful life events [36, 43, 45, 47, 77, 79, 94]	7
	IPV [44, 85, 90, 94]	4
	Poor SES [25, 33, 42]	3
	Poor housing [45, 53, 95]	3
	Lack of maternity leave [62, 83, 97]	3
	Partner education [53, 97]	2
	Poor self-efficacy [36, 42]	2
	Child development problems [36]	1
	Experience of childhood adversities [97]	1
Mental	Past history of depression [36, 43, 47, 50, 56, 62, 64, 68, 69, 85, 88, 95, 97]	13
	Anxiety and stress in the antenatal period [40, 43, 57, 60, 61, 68, 79, 90, 97]	9
	Antidepressant use during pregnancy [36, 43]	2
	Family history of mental health difficulties [57, 77]	2
Physical health	Smoking and alcohol history [43, 48, 57, 61, 64, 65, 69, 71, 76, 77, 91, 94, 95, 97]	14
	Poor baby weight [46, 54, 77, 79]	4
	Preterm birth or lower gestational age [46, 69, 85, 88]	4
	Very low BMI or obesity [46, 48, 65]	3
	Insomnia [43, 59]	2
	Poor general health [37, 62]	2
	Delivery complications [54, 88]	2
	Severe pregnancy symptoms [40, 45]	2
	Gestational DM [50]	1
	Lack of exercise [46]	1
	Poor antenatal healthcare [62]	1
	Chronic illness [90]	1
	Gestational hypertension [71]	1
	Somatic illness [43]	1
Patient characteristics	Low education [24, 33, 36–38, 40, 43, 48, 50, 61, 62, 65, 76, 79, 95]	15
	Young age [24, 33, 36, 38, 40, 46, 48, 50, 53, 77, 96]	11
	More children [38, 43, 54, 61, 62, 64, 65, 69, 77, 96]	10
	Old age [53, 60]	2

Among biological variables, a smoking history, alcohol consumption, and inadequate BMI were essential. Child developmental difficulties, poor baby weight, preterm birth, and delivery complications were also significant risk factors. Younger and poorly educated women having high parity were also at risk of developing more severe forms of PND.

Four studies provided risk factors for a worse prognosis while accounting for the onset period [77, 79, 82, 97]. As per these, those at high risk of developing postpartum depression had higher anxiety scores during the third trimester of pregnancy, high levels of stress in family, low education, minority ethnic groups and reported lower weight among their offspring [79]. Oh et al. reported no association of depression trajectories with age at childbirth, education level, delivery mode, breastfeeding, alcohol consumption, or smoking [82]. Van der Waerden et al. reported that French women who developed severe depression during pregnancy belonged to ethnic minority groups, lacked social support, had a history of mental health problems, pre-pregnancy substance use, and reported comorbid anxiety during pregnancy [85–87, 98]. Women with depressive symptoms persistent during their child’s preschool period only report their partner’s low educational level, pre-pregnancy mental, health treatment, and anxiety during pregnancy. While those with persistent depression reported experience of life events during pregnancy, work over-investment, pre-pregnancy mental health history/ treatment, and anxiety during pregnancy [85–87, 98].

### Child outcomes associated with PND subtypes with the worst prognosis

Child health outcomes were reported in 29 of these studies (Table 3). These studies reported several health problems among offspring born to mothers with complex depression trajectories: developmental problems, behavioural problems, poor health at delivery, and poorer physical health.

**Table 3:** Poor child outcomes associated with more severe and debilitating trajectories of PND

Domain	Condition
Developmental delay	Poor cognitive development [38] Poor motor development [38] Poor expressive language [26]
Behavioural problems	Conduct problems [41, 42, 51, 63, 85, 86, 99] Emotional problems [42, 52, 85, 86, 99] Externalizing and internalizing behaviours in both adolescence and adulthood [80] Internalizing behaviours [77, 78] Externalizing behaviours [77, 78, 89] Substance use [51] Social functioning [66, 69] Bullying victimization [63] Poor peer relationships [66, 86, 87, 99] Depression in adolescence [41, 77, 78] Poor academic performance [41, 80] High-risk internet use [82] Childhood trauma [89] Emotional dysregulation [93] Suicidal ideation [95] Poor postpartum bonding [27]
Delivery related complications	Preterm birth [36], low birth weight [52]
Physical illnesses	Chronic illnesses [49], wheezing [84], eczema [84], short stature [33]

Four studies found significant associations between delivery-related complications and physical illnesses among women with severe PND. These included preterm birth [36], low birth weight [52], low BMI, and low-fat mass [65] among children and adolescents. Among these studies, Farías-Antúnez et al. utilized Pelotas 2004 Birth Cohort [65], following perinatal women up to 11 years postpartum. After adjusting for confounders, children raised by mothers with chronic high PND have adequate body composition indices (BMI and fat mass). In contrast, Surkan et al. found that mothers with moderate to severe PND symptoms had children with shorter stature, and this persisted throughout the child’s first six years of life [33]. In addition, Puosi et al. reported a high risk of wheezing and eczema among children born to mothers with high (persistent or otherwise) depressive symptoms [84].

Lee and Park [38] reported that children born to mothers with aggravated depression reported significantly poorer cognitive (Cohen’s d = 0.43) and motor development (Cohen’s d = 0.34) according to Bayley’s Scale. Aoyagi et al. also found lower expressive language scores among children with mothers reporting late-onset PPD with a monotonic decline between 10 to 40 months postpartum [26].

A higher risk of developing conduct [41, 42, 51, 63] and emotional problems [52] was associated with severe forms of PND. Netsi et al. utilized the ALSPAC birth cohort data and found that children born to mothers with persistent PND were significantly at higher odds of developing behavioural problems (OR 4.84; 95%CI, 2.94–7.98); lower GCSE mathematics grades (OR = 2.65, 95% CI, 1.26–5.57) and higher depression rate (OR = 7.44, 95% CI, 2.89–19.11). Flouri & Loakeimidi utilized data from the UK Millenium Cohort Study to model the effect of trajectories of maternal depressive symptoms on antisocial behaviour and delinquency among children (aged three to 11 years, *N* = 12,494) [51]. They found that the boys born to mothers with chronically high or accelerating maternal depressive symptoms were more likely to report engaging in loud and rowdy behaviour, alcohol use, and bullying. Females exposed to chronically high maternal depressive symptoms were more likely to support the view that alcohol use is harmless [51]. Hammerton et al. reported that compared to offspring of mothers with minimal symptoms, the most significant risk of suicidal ideation was found for offspring of mothers with chronic-severe symptoms [95]. Only one study found no association of PND trajectories with alcohol use, abuse, and involvement in fights among postpartum Brazilian mothers in Pelotas, Brazil [64]. Fransson et al. found that postpartum bonding mediated most of the adverse effects of postpartum and persistent depression on child behaviour [27].

## Discussion

### Summary

This systematic review presents evidence on heterogeneous clinical presentations of PND. The reviewed studies employed different analytical approaches to visualize heterogeneous longitudinal trajectories, the pattern of symptom profiles, and symptom networks. Studies delineating trajectories reported both stable linear as well as unstable quadratic patterns. These trajectories ranged from minimal PND symptoms to stable linear and clinically severe, chronic, and persistent trajectories. Important symptom profiles challenged the notion of PND being considered a homogeneous latent construct. Most studies reported severe and persistent PND symptoms among perinatal women facing sociocultural stressors, less stable social networks, poverty, and displacement. At the same time, these were poor and persistent neurodevelopmental, socioemotional, and physical health outcomes among their children. The research evidence generally was robust, primarily obtained from extensive birth cohort studies or secondary analyses of RCTs.

### Implications for future research and clinical practice

This systematic review highlights an important consideration. Most studies reported a subset of perinatal women with either linear and worsening trajectories or persistent and severe symptom trajectories. It also highlighted the subset of women who are always at a high risk of relapse and recurrence. Moreover, this review demonstrates that this subset of perinatal women suffers from a double burden of severe PND symptoms and social, economic, and cultural diversities. This is an important finding because most mental health systems recommend utilizing cost-efficient stepped-care approaches [100, 101]. This is detrimental to the health of women who are not the right candidates for these stepped-care approaches and require high-intensity treatment at the outset to ease their suffering and offset poor infant and child outcomes.

This systematic review presents insights into the nature of PND. First, it demonstrates the heterogeneous presentations of PND and the associated differential risk factors. This challenges the prevailing understanding of PND as a homogenous latent construct based on the principles of essentialism and reductionism in psychiatry and psychometric research [102, 103]. There is, however, currently a lack of pathophysiological research presenting different mechanisms underpinning different heterogeneous profiles of PND [35]. Furthermore, more basic and clinical research is needed to understand this, accounting for this heterogeneity. This is vital because treating PND as a homogenous construct has hindered meaningful empirical research into the aetiology of PND [14]. Furthermore, although PND is considered a discrete diagnostic category, it has significant overlaps with symptoms of other disorders, including somatic and hypochondriac illnesses and anxiety [25], which usually bear poor prognosis.

Building on the evidence from this systematic review, it is crucial that interventions for PND be provided as early as possible. Although the current WHO recommendations [104, 105] currently emphasize acting during the early antenatal period, distinct trajectories of PND have also been shown with onset in early or postpartum. Therefore, we opine that screening and prevention should start earlier when the pregnancy is planned and during the early postpartum period. For instance, such screening and prevention strategies should be planned for those with prodromal symptoms (high distress pre-pregnancy) or those considered high-risk due to socioeconomic factors and pre-existing psychiatric and clinical pathologies [87], as this subgroup is at the highest risk for developing severe PND trajectories. This notion is also supported by Phua et al. who observed that the symptom networks among postpartum women are highly interconnected as they keep reinforcing each other over time and thus, become harder to treat [34].

This review has several clinical implications. Firstly, we opine that these findings can be used to develop cost-effective screening and prevention and treatment referral pathways. These pathways can be envisaged from a population health paradigm from promotion and prevention to treatment. From a health promotion perspective, policy makers must recognize the psychosocial risk factors that predispose perinatal women to severe forms of PND. As noted in this review, women with severe PND experience racism as migrant women, poverty, gendered oppression, and the lack of a social safety net. There is a need to address the intersecting effects of these social risks on perinatal mental health. Therefore, stakeholders must consider the perinatal experience's psychosocial underpinnings and design relevant policies [106]. Interdisciplinary approaches are required to bring about sustained health effects among the perinatal populations. For instance, using developmental economics approaches to tackle inequalities among perinatal women facing adversities can positively affect overall well-being [107]. These approaches have shown great promise in uplifting the general population's quality of life and mental health in recent trials [107].

For prevention, community-based surveys employing PND screening tools and social risk questionnaires [104, 108] can be utilized to identify women with either high social risk or prodromal depressive symptoms. Those found to be at the highest risk can be offered low-intensity interventions as recommended by the WHO and the US Preventive Services Taskforce [105, 109]. The same surveys could also be used to screen women who have already developed PND. In this context, these social risk questionnaire and depression screening tools can help identify heterogeneous trajectories of PND. This is an invaluable strategy and can lead to the development of next-generation of prognostic tools [110], which can match patients with PND to either low-intensity or high-intensity care depending on the severity and psychosocial risk; thus, helping improve the prognosis of women suffering from PND.

Another critical consideration in the prognosis of PND is the presence of overlapping anxiety symptoms [25]. There is considerable evidence that comorbid anxiety symptoms among women with PND worsen their prognosis [110]. Therefore, screening, prevention, and treatment strategies for PND must be considered. Using such transdiagnostic approaches has also been recommended in the Lancet’s Commission for Global Mental Health and sustainable development [111]. Furthermore, in this context, assessment of PND among women calls for either use of elaborate psychometric scales including symptoms of anxiety [112] or pairing depression-focused scales (such as the Patient Health Questionnaire) with scales for assessment of anxiety [113, 114].

The majority of evidence presented in this review pertains to the longitudinal trajectories of PND, with a focus on the severity of symptoms over time. While the timing of PND onset is a critical aspect of this discussion, it's important to note that different trajectories may not necessarily indicate a fundamental heterogeneity in the condition itself. We propose that variations in trajectory patterns could be influenced by factors such as life events, socioeconomic conditions, and access to treatment services (Table 2). These differences in baseline characteristics could contribute to the diverse trajectories of perinatal depressive symptoms observed across studies. This further supports our proposition that variations in trajectory patterns may be partly explained by psychosocial differences among women.

However, it's also possible that part of this variation could be attributed to differences in the timing of symptom onset or the specific symptom profiles. For instance, Waqas & Rahman [25] demonstrated a steep decrease in PND severity scores among perinatal women with somatic PND. This connection between diverse symptom profiles and varying longitudinal trajectories has not been extensively explored, and we recommend that future research investigates PND trajectories among perinatal women with different symptom profiles.

### Strengths & limitations

This systematic review presents updated evidence on heterogeneous profiles of PND. It provides a comprehensive overview of different types of empirical research in this domain and summarizes the risk factors associated with subtypes of PND with the worst prognosis. Moreover, evidence is also presented for the intergenerational effects of PND. The studies included in this review were generally of high quality and utilized datasets from large cohorts. However, large disparities in research evidence were noted, with only a small subset from LMICs. Such research delineating longitudinal trajectories and symptom profiles requires extensive infrastructure such as that of established birth cohorts in the UK [92]. Brazil contributed the most significant number of studies among the LMICs, where almost all utilized the dataset from the Pelotas birth cohort [62–67, 69, 70, 75, 115, 116]. Therefore, we recommend that investigators based in LMICs build birth cohort infrastructure to delineate the nature of PND, associated risk factors, and inequities in their communities. This will help formulate data-backed policies in perinatal mental health and help intelligent channelling of resources to screen, prevent, and treat perinatal mental disorders [117].

Another limitation of this review is that while we have synthesized the heterogeneous symptom profiles and longitudinal trajectories of perinatal depression, we have not conducted a detailed meta-synthesis of the risk factors associated with trajectories of worse prognosis and long-term sequelae. This task would involve the meta-aggregation of heterogeneous effect sizes, as well as a quality review of studies using scales for epidemiological studies of risk factors and long-term consequences. Given the complexity and scope of this task, it was beyond the purview of our current review. However, we recognize the importance of this endeavour for a comprehensive understanding of PND and suggest it as a direction for future research. This limitation does not diminish the value of our current findings but rather highlights an area where further work is needed to enhance our understanding of PND.

### Supplementary Information

**Additional file 1:** **Table S1.** Details of studies presenting heterogeneous symptoms profiles of PND. **Table S2.** Details of studies presenting longitudinal trajectory of PND symptoms based on severity and chronicity. **Table S3.** Study details for longitudinal trajectory of PND symptoms based on time of onset.

**Additional file 2:** **Supplementary file 2.** Quality of reporting in included studies as per the Guidelines for Reporting on Latent Trajectory Studies (GRoLT).

## Data Availability

All data associated with this manuscript are available as Supplementary files.
