# Supplementary material for: Exploring Heterogeneity in perinatal depression: a comprehensive review
Source: BMC Psychiatry. 2023 Sep 4;23:643. doi: 10.1186/s12888-023-05121-z (PMC10478465; doi:10.1186/s12888-023-05121-z)
Supplement: Supplementary file 1 — Additional file 1: Table S1. Details of studies presenting heterogeneous symptoms profiles of PND. Table S2. Details of studies presenting longitudinal trajectory of PND symptoms based on severity and chronicity. Table S3. Study details for longitudinal trajectory of PND symptoms based on time of onset. [file 12888_2023_5121_MOESM1_ESM.docx]

Supplementary Table 1: Details of studies presenting heterogeneous symptoms profiles of PND

| **Study ID** | **Sample size** | **Statistical technique** | **Criteria for choosing number of classes** | **Country** | **Time of recruitment** | **Data collection waves** | **Scale for subtyping or trajectory** | **Number of classes** | **Names of heterogeneous classes** | **Freq of heterogeneous classes** |
| --- | --- | --- | --- | --- | --- | --- | --- | --- | --- | --- |
| Eastwood , 2021 | 9646 | Latent class analysis | AIC, BIC | Australia | Pregnancy and postnatal | 1 or 2; antenatal booking visit and/or postnatal EPDS scores | EPDS | 4 | 1.Locals Group L, 47%, n = 4573); 2. Stressed locals (Group Ls, 4%, n = 353); 3. Migrants (Group M, 48%, n = 4622); 4. Stressed Migrants | 1. 4573 2. 353 3.4622 4. 98 |
| Putnam , 2015 | 17912 | Latent class analysis | i) entropy value ii) Vuong-Lo-Mendell-Rubin likelihood ratio value iii) Bayesian information criterion iv) Akaike’s information criterion, and v) bivariate residuals | Multiple countries | All | NA | EPDS/HAMD-17 | 3 | Class 1: Not depressed or anxious \| Class 2: feeling symptomatic in terms of sadness, blaming themselves unnecessarily, and having difficulty sleeping \| Class 3: In addition to symptoms of class 2, had notably more severe symptoms than those in class 2 for feeling panicky, sad, and crying often, and particularly for thoughts of harming oneself often | Class 1= 3484 \| Class 2= 2342 \| Class 3= 730 |
| Phua , 2020 | 1119 | Network analysis | NA | Singapore | Pregnancy | NA | BDI/EPDS/STAI |  | 1. Connections among the symptoms will become stronger as they keep reinforcing each other over time. 2. Qualitative differences in core depressive-anxiety symptoms across the peripartum periods. 3. During pregnancy, the cognitive-affective symptoms were more central. 4. These symptoms were related to feeling worthless, agonizing over past failures, or having disturbing thoughts. 5. After pregnancy, the sense of being overwhelmed or being punished were most central in the depressive-anxiety network. 6. During pregnancy, the top bridging symptoms were related to feelings of guilt, nervousness, or being a failure. After pregnancy, the top bridging symptoms were related to self-blame, feeling overwhelmed, and excessive worries. |  |
| Putnam , 2017 | 663 | K means cluster analysis | cubic clustering criterion | Multiple countries | Pregnancy | NA | EPDS | 5 | 1. severe anxious depression 2.moderate anxious depression 3.anxious anhedonia 4.pure anhedonia 5.resolved depression. | 1. 211 2.123 3.79 4.75 5.175 |
| Putnam , 2015 | 10801 | Latent class analysis | BIC, entropy, Vuo-Long-Mendell-Rubin likelihood ratio | Multiple countries | Pregnancy | NA | EPDS and Hamilton | 3 | Class 1, Class 2, Class 3 | Tier 1: Class 1(no depressed)=3484 Class 2 (symptomatic)=2342 Class 3 (severe depression)=730 Tier 2: Class 1 (no depressed)=759 Class 2(symptomatic)=2099 Class 3 (severe depression)=1387 |
| Saldaña , 2021 | 587 | Exploratory factor analysis (EFA) + confirmatory factor analysis (CFA) | NA | USA | Postpartum | NA | BDI-II and Postpartum Depression Screening Scale (PDSS) | 5 factor model | 1.anxiety/thought disorder 2. cognitive depression 3.suicide 4.somatic/neurovegetative 5. sleep |  |
| Santos , 2017 | 515 | Gausian Graphical Model+ Proof of principle analyses |  | USA | Pregnancy |  | CES-D |  | Cortisol was the biomarker most often connected to symptoms, showing a positive relationship with positive mood (happiness) and social-related symptoms (loneliness, effort, and unfriendly). There was also a negative association between cortisol and two other mood symptoms: feeling blue and crying. |  |
| Sun , 2019 | 2783 | Latent class analysis | AIC, BIC, sample size adjusted BIC, Vuong‐Lo‐Mendell‐Rubin likelihood ratio test, bootstrapped likelihood ratio test, clinical interpretability | China | All | 1 | PHQ-9 | 5 | Class 1:moderate‐to‐severe symptoms group Class2: severe symptoms group; Class 3: severe physio‐somatic symptoms and moderate anhedonia group; Class 4: mild physio somatic symptoms group; Class 5: no symptoms group | Class 1=306, = Class 2=280, Class 3= 726, Class 4= 937, Class 5= 534 |
| Surkan , 2014 | 10700 | Unadjusted loigstic regression, chi-square | AIC | USA | Postpartum | 1; 9 months | CES-D | 3 | 1. No symptoms 2. Mild symptoms 3. Moderate/severe symptoms |  |
| Waqas , 2021 | 903 | Exploratory factor analysis + two-step cluster analysis | AIC (log-likelihood distance measure) | Pakistan | Pregnancy | 3; Baseline, 6 months, 12 months | DSM IV, Hamilton Depression Rating Scale | 4 | Cluster 1 (Atypical depression), Cluster 2 (Somatic symptoms) Cluster 3(Mild depression) Cluster 4 (Mixed anxiety and depression) | 88, 242, 377, 196 |
| Aoyagi , 2019 | 1258 | Growth curve analysis | NR | Japan | pregnancy | 10,14,18,24,32,40 months | EPDS | 3 | No PPD, early onset PPD, late onset PPD | no PPD=823 early=103 late=43 |
| Fransson , 2020 | 2663 | Linear regression modelling | NR | Sweden | Pregnancy | 4; gestational weeks 17, 32, and postpartum week six and month six | EPDS, MINI, DSRS scores | 4 | 1. No depressive symptoms 2. Antenatal depression 3. Postpartum depression 4. Persistent depression | 1. 870 2. 114 3. 152 4.217 |
| Tebeka , 2021 | 3310 | Logistic regression | AIC, penalised likelihood ratio approach (LASSO) | France | Pregnancy | 2; 8 weeks after pregnancy, 1 year after pregnancy | Diagnostic; DIGS DSM V | 2 | 1. Early onset PPD 2. Late onset PPD | Early=250 Late=235 Controls=1859 |

Supplementary Table 2: Details of studies presenting longitudinal trajectory of PND symptoms based on severity and chronicity

| **Study ID** | **Sample size** | **Statistical technique** | **Criteria for choosing number of classes** | **Country** | **Time of recruitment** | **Timepoints for data collection** | **Variables** | **Number of trajectories** | **Names of trajectories** | **Explain trajectory pattern** | **Freq of trajectory** |
| --- | --- | --- | --- | --- | --- | --- | --- | --- | --- | --- | --- |
| **Studies reporting two trajectory patterns** | | | | | | | | | | | |
| Kingsbury , 2015 | 6753 | K means cluster analysis | NR (uncertain, please double-check) | Australia | Pregnancy | 4; 6 months, 5, 14 and 21 years | DSSI | 2 | 1.No/stable low 2.Escalating | No/stable low group= DSSI scores around M=0.42 at 6 months, and fluctuate between .35-.38 throughout 21 years. Escalating depression group=DSSI scores start at M=1.34 and esclate steadily throughout 21 years, to M=3.45 at 21 years | 1. 2361 2. 630 |
| Hong , 2022 | 550 | Latent growth mixture modelling | AIC, BIC, BLRT, VLMR, entropy | China | Pregnancy | 3; late pregnancy (32±4weeks of gestation)and 1 and 6 weeks postpartum | EPDS | 2 | 1. Increasing 2. Decreasing | decreasing= had an estimated mean baseline score of 7.09, decreased to 5.64 at one-week postpartum and stabilized at at six-weeks postpartum. increasing group= characterised by no depressive symptoms at recruitment (estimated mean=6.90), but increasing depression scores at one- and six-weeks postpartum | 1. 26 2. 524 |
| Giallo , 2014 | 5107 | Latent growth modelling | BIC, AIC, entropy, Vuong–Lo–Mendall–Rubin likelihood ratio test | Australia | Postpartum | 4; child age 3–12 months, 2–3 years, 4–5 years, and 6–7 years | K-6 | 2 | 1. Minimal depressive symptoms 2. Persistently high depressive symptoms | class 1= characterised by low average distress scores (K6 score of 2.52) in the postnatal period , which remained consistently low across the childhood period and decreased at each wave. Class 2= higher average distress scores in the postnatal period which remained stable across the early childhood period | 1. 4100 2.779 |
| Lee , 2020 | 737 | Growth mixture modelling (LCGA) | AIC, sample size-adjusted BIC adjusted Lo–Mendell–Rubin Test, Entropy | Malawai | Pregnancy | 3; Pregnancy, 1 year after birth, 2 years after birth | PHQ-9 | 2 | 1. Depression aggravated group 2. Depression mitigated group | In depression mitigated group, depression dcereased in the years from pregnancy till 2 years pospartum, in depression aggravated group, depression increased | 1. 70 2. 647 |
| **Studies reporting three trajectory patterns** | | | | | | | | | | | |
| Madigan , 2017 | 501 | growth mixture modelling | AIC, BIC, aBIC, entropy, LMR-LRT | Canada | Postpartum | 4; 2 months, 18 months, 36 months, and 54 months | CES-D | 3 | 1. Low stable 2.High-decreasing 3. Moderate increasing | Low-stable: scores in the low-stable class consistently remained below the clinically depressed range (⩽16) on the CES-D. High-decreasing class= scored well above the clinical cut-off at T1, at the clinical cut-off at T2, and then dropped below the cut-off at T3 and T4. moderate-increasing group= had average initial depression levels at the clinical cut-off, and these levels increased well into the clinical range by the final assessment poi | 1. 84% 2. 9.5% 3. 6.5% |
| Kiviruusu , 2020 | 1670 | Latent profile analysis | AIC, BIC, VLMR, LMR-A, entropy | Finland | Pregnancy | 4; gestational week 32 and 3, 8 and 24 months postpartum | CES-D | 3 | 1. Low 2. Moderate 3. High | The largest profile was the “low” trajectory group constantly reporting (on average) mild depres The second largest group was the “moderate” trajectory with stable moderate or subthreshold depressive symptoms (mean CES-D above 6 and below 9 points). The “high” profile reported (on average) constantly depressive symptoms above the clinical threshold (CESD>10 symptoms (mean CES-D10). | low=1053 moderate=470 high=147 |
| Simons , 2020 | 623 | Group based trajectory models | AIC, BIC, adjusted BIC,log likelihood test | USA | Pregnancy | 4; T1= 1st-2nd trimester T2=3rd Trimester T3=6 months postpartum T4= 12 months postpartum | CES-D | 3 | 1. Stable low 2. Moderate declining 3. Stable High | Stable low: low mean CES-D scores at each time point moderate declining: started above the cutoff score of 16 at baseline, hovered around the cutoff at T2, and declined below the cutoff score in the postpartum period\|stable high: depressive symptom scores well above the cutoff score across all time point | Stable low= 376 Moderate declining= 184 Stable High= 63 |
| Kingsbury , 2018 | 6753 | Latent class growth modelling | BIC, average posterior probability | Australia | Pregnancy | 7; first clinic visit, six months, five years, 14 years, 21 years, 27 years, post birth | DSSI | 3 | 1. Low 2. Middle 3. High | NR | 1. 1179 2.1018 3.238 |
| Kingsbury , 2018 | 6753 | semi-parametric mixture methods | BIC, posterior probability score, average group membership socre | Australia | Pregnancy | 6; First clinic visit, 6 months, 5 years, 14 years, 21 years, and 27 years after birth, | DSSI | 3 | 1. Low stable 2. Moderate-stable 3. Moderate-rising | low stable= DSSI scores between 0-1 throughout 27 years, around 0.5 at FCV and then decreasing over time to almost 0 at 27 moderate stable=dssi scores near 2 at FCV, hovering between 1 and 2, to below 1 at 27 years Moderate-rising= DSSI scores higher than moderate-stable, between 3 and 4 at FCV then steadily icnreasing between birth and 21 years where they peaked around DSSI=6, then decreasing to around 4.5 at 27 years | 1. 49.1% 2.42.3% 3.8.6% |
| Boekhorst , 2019 | 1832 | Growth mixture modelling | BIC, entropy, fullinformation maximum likelihood estimates,Lo-Mendell-Rubin Likelihood Ratio Test, and Bootstrapped Likelihood Ratio Test | Netherlands | Pregnancy | 3; 12, 22, and 32 weeks of pregnancy | EPDS | 3 | 1. Low stable 2. Decreasing 3. Increasing | Low stable=women showed a stable and low intensity pattern of depressive symptoms with mean E(P)DS scores of less than 4 during pregnancy.Decreaaing= showed a pattern reflecting a high level of depressive symptoms in early pregnancy, which decreased over time: in short, the ‘decreasing’ pattern. At the first trimester, the mean E(P)DS score was 13.6, which decreased significantly to 6.8 at the last trimester. Increasing= women had a moderately high mean E(P)DS at first trimester (8.7) that increased significantly towards the end of pregnancy (13.2) | 1. 1517 2. 128 3. 187 |
| Miller , 2022 | 10038 | Not applicable. (The Edinburgh Postpartum Depression Scale score trajectories were categorized as improved, stable, or worsened based on whether the scores changed by at least 1 standard deviation between the 2 visits) | NR | USA | Pregnancy | 2; between 6-14 weeks after gestation and 22-30 weeks gestation | EPDS | 3 | 1. Improved 2. Stable 3. Worsened | These trajectories were defined by a change in the EPDS score between visit 1 and visit 3 by at least 1 standard deviation.Of women who completed EPDS screens at 2 time points, 1141 (13.0%) had improved, 6663 (75.9%) had stable, and 980 (11.2%) had worsened depressive symptoms. Of the 980 women with worsened depressive symptoms, only 277 (28%) had an EPDS score of =>13 | 1. 1141 2. 6663 3. 980 |
| **Studies reporting four trajectory patterns** | | | | | | | | | | | |
| Sutter-Dallay , 2012 | 579 | Semiparametric mixture model | BIC, average posterior probability | France | Pregnancy | 8; 8 months of pregnancy, at days three, six weeks, 3, 6, 12, 18 and 24 months after delivery. | CES-D | 4 | 1. Never 2. Postpartum 3. Antepartum 4. Chronic | “Postpartum”, depressive symptoms during the third trimester of pregnancy is the lowest of the sample, which tends to increase rapidly and to reach a maximum 13.7 months after birth. “Never”, shows an average level of depressive symptoms during the last trimester of pregnancy. “Antepartum” starts with a high average level of depressive symptoms during pregnancy which tends to decrease until the 13th month after delivery. This group is also characterized by a very slight increase after 24 months. “Chronic”, includes subjects with a stable and high mean level of depressive symptoms from the end of pregnancy to 2 years after birth. | 1. 419 2.22 3.122 4.16 |
| Ferro , 2015 | 1534 | Latent Class Growth Modelling | BIC, posterior probability score | Canada | Postpartum | 3; child age 10 –11, 12–13, and 14 –15 years | CES-D | 4 | 1. low 2.decreasing 3.increasing 4.high | The first trajectory identified was one of consistently low levels of depressive symptoms (low). Over 80% of mothers (n=9710) were in this trajectory group, with a mean CES D score of 2.7 when youths were 10 –11 years and 2.6 at 14 –15 years. The second trajectory included 8.2% (n =969) of mothers and had decreasing symptoms of depression from 14.2 to 5.9. The third trajectory (n 838, 7.1%) described mothers who had increasing symptoms that went from 6.4 to 15.9 during the follow-up. The fourth trajectory identified mothers with consistently high levels of depressive symptoms (n= 295) | low= 9710, decreasing=969, increasing= 838, high=295 |
| Choi , 2022 | 899 | Growth mixture modelling | Akaike’s Information Criterion (AIC), Bayesian Information Criterion (BIC), Entropy, Bootstrapped likelihood ratio test (BLRT) and Vuong–Lo–Mendell–Rubin Likelihood Ratio (VLMR) | USA | Pregnancy | 4; first or second trimester of pregnancy, third trimester of pregnancy, month 1 (0–30 days) postpartum, months 2 to 6 (31–180 days) postpartum. | EPDS | 4 | 1. Low stable group 2.Increasing group 3. Decreasing group 4. High-stable group | 1) Low-stable group, i.e., persistently low prenatal and postpartum EPDS scores 2) Increasing group, i.e., low EPDS scores at first prenatal screen, with scores increasing over time and exceeding the cutoff for likely postpartum depression 3) Decreasing group, i.e., EPDS score above the cutoff prenatally but decreasing to below the cutoff for likely postpartum depression 4) High-stable group, i.e., persistently high prenatal and postpartum EPDS scores | 1. 703 2. 36 3.63 4.94 |
| Ahmed , 2019 | 646 | Semiparametric, group-based approach for modeling developmental trajectories | BIC, Bayes factor | Canada | Pregnancy | Early pregnancy, late pregnancy, early postpartum, 36 months postpartum, 60 months postpartum | EPDS | 4 | 1. low stable \| 2. Moderata stable \| 3. Moderate increasing \| 4. High decreasing | 1= EPDS scores were consistently low throughout the follow-up period \| 2= moderate level of depressive symptoms across the period of follow-up that minimally decreased over time \| 3= moderate-increasing group were slightly higher than those of the moderate-stable group during pregnancy; however, their EPDS scores increased significantly postpartum \| 4= high levels of depressive symptoms during pregnancwhich started to decrease gradually after giving birth, except for a slight increase between the third and fifth year | 1= 215 \| 2=332 \| 3=32 \| 4= 36 |
| Dekel , 2019 | 824 | Growth mixture modelling | BIC and sample-sized adjusted BIC, entropy , the Lo-Mendell-Rubin (LRT) and bootstrap likelihood ratio test (BLRT) | Finland | Pregnancy | 3; first trimester (6–12 gestation weeks), third trimester (28–43 gestation weeks) and again in the early postpartum period (at 8 weeks postpartum) | EPDS | 4 | 1. Chronic 2. Delayed onset 3. recovered 4. resilient | Most women were resilient to PDD. A small number of women had a chronic state of PDD that began prior to delivery and continued postpartum (chronic, class 3). The forthcoming delivery evoked PPD in some women, which continued postpartum (delayedonset, class 2), and for others symptoms improved drastically over time (recovered, class 4). | 1. 9 2. 83 3.55 4.677 |
| Kingston , 2018 | 1983 | Longitudinal latent class analysis | Likelihood ratio statistic (L2), AIC, BIC, Vuong-Lo-Mendall-Rubin Likelihood Ratio Test | Canada | Pregnancy | 4; 1) at <25 weeks’ gestation; 2) 34–36 weeks’ gestation; 3) four months postpartum; and 4) one year postpartum | EPDS | 4 | 1. Minimal depressive symptoms 2. Early postpartum depressive symptoms 3. Subclinical depressive symptoms 4. High depressive symptoms | Minimal depressive symptoms= largest trajectory consisted of women who reported “minimal depressive symptoms” from pregnancy to one year postpartum (n = 1283, 64.7%). Early postpartyum symptoms= The second trajectory, with a peak in the mean EPDS slightly higher than the sub-clinical group, consisted of women who reported “early postpartum depressive symptoms” Subclinical symptoms= trajectory consisted of women who reported “subclinical depressive symptoms” over time. High depressive symptoms= women who reported “high depressive symptoms” over time | 1. 1283 2. 216 3. 372 4. 112 |
| Lim , 2019 | 2820 | Group based trajectory models | BIC, average posterior probability, %age membership in trajectory based on previous local studies | Singapore | Pregnancy | 4; first trimester ,second trimester , third trimester , and prior to delivery (after 34 weeks of gestation) | EPDS | 4 | 1. Persistently no 2.Persistently mild 3.Persistently low threshold 4. Persistently moderate | trajectory 1= linear, "persistently no"; had depressive symptoms (EPDStotal = 3.18–4.14), with participants endorsing a score of 0 (“Never” or “Not at all”) for a majority of the 10 items. Trajectory 2 was cubic and relatively non-fluctuating, and named “persistently-mild” depressive symptoms (EPDStotal = 7.04–8.16). Trajectory 3 was linear and named “persistently- subthreshold” depressive symptoms, as it was consistently below the recommended EPDS cutoff of 14/15 (EPDStotal = 10.70–12.03). Trajectory 4 was linear and named “persistently-moderate” depressive symptoms as scores were consistently above the recommended EPDS cutoff of 14/15 (EPDStotal = 15.75–16.40), suggesting that these women may have consistently experienced probable clinical depression throughout the pregnancy | 1. 339 2.306 3. 197 4.84 |
| Putnick , 2020 | 4866 | Linear latent growth model | full information maximum likelihood estimation (FIML) | USA | Postpartum | 4, 12, 24, and 36 months postpartum | EPDS | 4 | 1. Low-stable 2. Low-increasing 3. Medium-decreasing 4. High-persistent | low-stable= characterized by low symptoms at all waves; low-increasing= characterized by initially low but increasing symptoms, medium-decreasing= characterized by initially moderate but remitting symptoms. high-persistent= characterized by high symptoms at all waves | 1. 3637 2.398 3.613 4. 218 |
| Denckla , 2018 | 12121 | Latent growth mixture modelling | AIC, BIC, sample size adjusted BIC, entropy, Vuo-Long Mendell Likelihood ratio test, BLRT, full-information maximum-likelihood (FMIL), log-likelihood ratio | UK | Pregnancy | 7; 18 and 32 weeks of gestation, 8 weeks after the birth of the baby, and thereafter at 8, 21, 33, and 61 months. | EPDS | 4 | 1. Resilient 2. Improving 3. Chronic 4. Emergent | Resilient= stable low symptoms throughout. Chronic= had individuals with high levels of depressive symptoms both during pregnancy and after childbirth, declining slightly over the 5 years after childbirth. Improving= had elevated levels of depressive symptoms during pregnancy that declined after the birth. Emergent= moderate levels of depressive symptoms during pregnancy followed by a sharp elevation after childbirth | Resilient= 7070 Improving= 701 Chronic= 974 Emergent-364 |
| Ladyman , 2021 | 856 | Latent class analysis | BIC, posterior probability scores | New Zealand | Pregnancy | 3; 35–37 weeks of gestation, 12weeks postnatal, 3 years postnatal | EPDS and K-10 scores | 4 | 1. Chronic High Maori women 2. Stable low Maori women 3. Chronic high non-maori women 4. Stable low non-maori women | Māori women in both trajectories were more likely than non-Māori women to have clinically significant depressive symptoms at every time point. All trajectories rapidly decreased in probability of having clinically depressed symptoms between 0-0.5 years after childbirth, after which all trajectories saw a steady increase in the probability till 3 years, the highest for maori-chronic high women, followed by maori-stable low, non-maori chronic high, and finally the lowest probability for non-maori stable low women | chronic high maori=57 chronic high non maori=77 |
| Flouri , 2018 | 15590 | Latent class analysis | BIC, AIC, Log-likelihood, ssa BIC, entropy | UK | Postpartum | 4; child ages 3,5, 7, 11 | K-10 | 4 | 1. Consistently low 2. Chronically high 3. Moderate accelerating 4. Moderate decelerating | consistently low included mothers with conistently low levels of symptoms throughout. Chronically high: ‘chronically high’ depressive symptoms throughout. The two intermediate classes included mothers with levels of symptoms that were moderate at the frst two time-points but either increased (‘moderateaccelerating’) or decreased (‘moderate-decelerating’) with time | 9133, 649, 924, 1799 |
| **Studies reporting five trajectory patterns** | | | | | | | | | | | |
| Mora , 2009 | 1735 | Growth mixture modelling | AIC, BIC, aBIC, entropy, log-likehlihood test | USA | Pregnancy | 4; prenatal, 3 months postpartum, 11 months postpartum, 25 months postpartum | CES-D | 5 | 1. Chronic 2. Antepartum 3. Postpartum 4. Late 4. Never | 1) chronic, persistently high level of depressive symptoms 2) antepartum depressive symptomatology present only at the first prenatal visit 3) postpartum depressive symptoms present within 6 weeks of delivery that subside over time 4) late with low levels of depressive symptoms ante- and peripartum that increase in the second year postpartum and 5) never elevated, with continuous low levels of depressive symptoms | 1. 114 2.110 3. 162 4.112 5.1237 |
| Jacques , 2020 | 3040 | Group based trajectory models | maximum BIC, posterior probability score | Brazil | Pregnancy | 4; antenatal, 3-, 12-, and 24-month visits | EPDS | 5 | 1. Low 2.Moderate Low 3. Increasing 4. Decreasing persistent 5. Chronic high | “Low” and "Moderate"= groups had an EPDS score < 10 at all-time points of analysis . Increasing= mothers exhibited a constant increase in EPDS scores over the period of analysis. Decreasing but persistent= included mothers, who presented a high depressive symptom score in the antenatal period, had a decrease at 3 months, and maintained a score around 10 between the 12- and 24- month time points. Chronic high= mothers, presented a high EPDS scores of 17 in the antenatal period, declining to 15 at 3 months, rising again to 19 at 12 months, and again decreasing to 17 at 24 months after delivery | 1. 1109 2.1219 3. 298 4.294 5.120 |
| Matijasevich , 2015 | 4321 | Semiparametric group based modelling + multivariable logistic regression | BIC, interpretability, posterior probability scores | Brazil | Postpartum | 5; 3, 12, 24 and 48 months and 6 years after delivery | EPDS | 5 | 1. low 2. moderate low 3.increasing 4. decreasing 5.high-chronic | women in “low" “moderate low”,had EPDS scores <10 across all time points suggesting low depressive symptomatology.“increasing” showed a consistent increase in depressive symptoms during the study period. “decreasing”showed high EPDS scores in the first two years postpartum and a marked decrease afterwards.“high-chronic” had high EPDS scores all through the study period | low= 1161 moderate low=1361 increasing=300 decreasing=329 high-chronic=181 |
| Azeredo , 2017 | 4231 | Semiparametric group based modelling + ordinal logistic regression | BIC, posterior probability score | Brazil | Postpartum | 3; Birth, 3 months, 11 years | EPDS | 5 | 1. “Low” depressive symptoms 2. ‘Moderate low” depressive symptoms 3.“Increasing” depressive symptoms 4.“Decreasing” depressive symptoms 5.“High chronic” depressive symptoms | Groups 1 (low) and 2 (moderate-low) had EPDS scores below 10 across all time points, suggesting low depresive symptomatology. Group 3 (increasing) had a consistent increase in depressive symptoms during the study period. The fourth group (decreasing) showed high EPDS scores in the first 2 years postpartum and a marked decrease afterwards. Group 5 (high-chronic) had high EPDS scores throughout the study period | 1161, 1361, 300, 329, 181 |
| Bozzini , 2021 | 4231 | Semiparametric modelling approach + Multivariate logistic regression | NR | Brazil | Postpartum | 6; at home: ages 3 months (0.1), 11.9 months, 23.9 months, 49.5 months and at a research clinic at 6.8 and 11.0 years | EPDS | 5 | 1. Low 2. Moderate Low 3. Increasing 4. Decreasing 5. Chronic High | “low” trajectory group is represented by a linear trajectory comprising women with EPDS< 10 across all time points. The “moderate low” group is represented by quadraric trajectory comprising mothers with EPDS<10 across all time points. The “increasing” group showed a consistent increase in depressive symptoms during the study period and the “decreasing” group showed high EPDS scores in the first 2 years postpartum and a marked decrease afterwards. “chronic high” trajectory demonstrated high EPDS scores all through the study period | 1. 1096 2.1417 3. 375 4.295 5. 164 |
| Farias-Antunez , 2020 | 4231 | Semiparametric group based modelling | maximum BIC, posterior probability score | Brazil | Postpartum | 6; 3 months, 12 months, 24 months, 48 months, 6 years, 11 years | EPDS | 5 | ‘Low’, ‘Moderate low’, ‘Increasing’, ‘Decreasing’ and ‘Chronic high’ | Group 1 (‘Low’) mothers scored 5 points or less in the EPDS throughout all evaluations; Group 2 (‘Moderate low’) mothers had EPDS scores lower than 10 points throughout the whole period; Group 3 (‘Increasing’) mothers included those with depressive symptoms that increased over time (mothers who scored <10 points until about 4 years into the postpartum period and thereafter presented increasing scores until reaching about 15 points at 11 years after childbirth); Group 4 (‘Decreasing’) mothers presented the opposite pattern (scoring between 10 and 15 points over the first Group 5 (‘Chronic high’) mothers scored over 13 points on the EPDS scale at every follow-up | ‘Low’= 1175 ‘Moderate low’=1411 ‘Increasing'=399 ‘Decreasing’=312 ‘Chronic high’= 170 |
| Maruyama , 2021 | 4321 | Semi-parametric group based modelling | maximum BIC, posterior probability score | Brazil | Postpartum | 6; 3.0 months, 11.9 months, 23.9 months, and 49.5 months, 6.8 years and 11 years | EPDS | 5 | 1. Low 2. Moderate Low 3. Increasing 4. Decreasing 5. High-Chronic | groups "low" and "moderate-low" included mothers with EPDS scores below 10 across all time points. "increasing" included women that had a consistent increase in depressive symptoms during the study period. "decreasing"was composed by women that showed high EPDS scores in the frst 2 years postpartum and a marked decrease afterwards. high-chronic included mothers with high EPDS scores across the 11 years of the study | 1. 1133 2.1466 3.385 4.316 5. 165 |
| Maruyama , 2019 | 4321 | Semi-parametric group based modelling | maximum BIC, posterior probability score | Brazil | Postpartum | 6; 3 months, 12 months, 24 months, 48 months, 6 years, 11 years | EPDS | 5 | 1. Low 2. Moderate Low 3. Increasing 4. Decreasing 5. Chronic High | groups 1 ("low") and 2 ("moderate-low") included mothers withlow depressive symptomatolofy EPDS scores below 10 across all time points. Group 3 ("increasing") included women that had a consistent increase in depressive symptoms during the study period. The fourth group ("decreasing") was composed by women that showed high EPDS scores in the first 2 years postpartum and a marked decrease afterwards. Group 5 ("high-chronic"), included mothers with high EPDS scores across the study period | Low and moderate low=2570, increasing=381, decreasing=316, high-chronic=168 |
| **Studies reporting > 5 trajectory patterns** | | | | | | | | | | | |
| McCall-Hosenfeld , 2016 | 3006 | semi-parametric mixture models | AIC, entropy, posterior probability scores | USA | Pregnancy | 4; third trimester (baseline) and at 1, 6, and 12 months postpartum | EPDS | 6 | Trajectory 1, trajectory 2, trajectory 3, trajectory 4, tracjetory 5, tracjetory 6 | trajectories were grouped from 1-6 depending on EPDS scores at baseline, with baseline scores being lowest in trajectory 1, highest in trajectory 6.all trajectory were stable or slightly decreasing with only trajetory 4 showing an increase. women who followed trajectory 4 were nondepressed at baseline with an EPDS of *7, similar to women belonging to trajectory 3, yet at 12 months they had an EPDS >15, similar to clinically depressed women in trajectory 6. | 1. 6.5% 2.42.2% 3. 36.5% 4.1.7% 5. 11.9% 6.1.3% |
| Netsi , 2018 | 15427 | Linear growth modelling | NR | UK | Postpartum | 8; 2, 8, 21, 33, 61, 73, 93, and 134 months after childbirth | EPDS | 7 | 1. Below threshold 2. Moderate but not persistent 3. Marked but not persistent 4. Severe but not persistent 5. Moderate persistent 6. Marked persistent 7. Severe persistent | a EPDS score of less than 13 points in the postnatal year. b EPDS score of 13 to 14 points at 2 months and less than 13 points at 8 months. c EPDS score of 15 to 16 points at 2 months and less than 15 points at 8 months. d EPDS score of 17 or more points at 2 months and less than 17 points at 8 months. e EPDS score of 13 to 14 points at 2 months and 13 or more points at 8 months. f EPDS score of 15 to 16 points at 2 months and 15 or more points at 8 months. g EPDS score of 17 or more points at 2 and 8 months | 75 |

Supplementary Table 3: Study details for longitudinal trajectory of PND symptoms based on time of onset

| **Study ID** | **Sample size** | **Statistical technique** | **Criteria for choosing number of classes** | **Country** | **Time of recruitment** | **Timepoints for data collection for trajectories** | **Variables** | **Number of trajectories** | **Names of trajectories** | **Explain trajectory pattern** | **Freq of trajectory** |
| --- | --- | --- | --- | --- | --- | --- | --- | --- | --- | --- | --- |
| Glasheen , 2013 | 829 | Growth mixture modeling+ logistic/multinomial regression analyses | Adjusted BIC, AIC, entropy, the Lo–Mendell–Rubin statistic | USA | Pregnancy | 5; 1st trimester, 2nd trimester, Delivery, 8 months, 17 months | CES-D | 2 | 1. Low pre and postnatal depression 2. High pre and postnatal depression | Low/high symptoms throughout all 5 assessment points with few decreases/changes | 1. 95 2. 482 |
| Barthel , 2017 | 776 | Growth mixture modelling | Akaike’s information criterion (AIC), Bayesian information criterion (BIC), Lo-Mendell-Rubin adjusted likelihood ratio test, bootstrap likelihood ratio test (BLRT), entropy, full information likelihood estimation | Coˆte d’Ivoire and Ghana | Pregnancy | 4; 3 months before and 3, 12, and 24 months after delivery | PHQ | 3 | 1. Asymptomatic 2.Recurrent risk 3.postnatal risk class | asymptomatic= stable low depressive symptoms during the last trimester of pregnancy and the 2 years following childbirth. The estimated mean PHQ-9 score of these women was 5.7 points at birth and remained low with a slightly decreasing tendency over time The recurrent risk class= average PHQ-9 score of 7.8 points at birth. Their depressive symptomatology decreased slightly and reached a score of 6.5 one year after birth. After that, the PHQ-9 score increased gradually up to 12.9 points 2 years after birth. The postnatal risk class had a mean of 11.4 PHQ-9 points at birth. PHQ-9 scores increased with a peak of 13.6 one year after birth. Afterwards, the depressive symptoms decreased and were at an asymptomatic level of 4.2 PHQ-9 points 2 years after birth | 1. 710 2.33 3.33 |
| Najman , 2017 | 6753 | Latent class growth modelling | BIC, posterior probabilities | Australia | Pregnancy | 6; First clinic visit, 6 months, 5 years, 14 years, 21 years, and 27 years after birth, | DSSI+ CIDI | 3 | 1. Low 2. Mild 3. High | low/no depression = very few if any symptoms of depression at the time of recruitment (FCV) and throughout 27 years. mid-trajectory= entered the study with a mean of about one symptom. little change in the mean number of symptoms of depression over the 27 years with the exception of a decline in the average number of symptoms from the 21–27-year follow-ups. high trajectory= highest mean number of symptoms prior to the birth and experiences an increasing mean number of symptoms of depression from the period shortly after the birth of the study child to the 14-year follow-up, and plateaus thereafter. average symptoms at the 21-year follow-up are about twice as high as they were at the FCV, with a mean of over three DSSI symptoms experienced by each participant at the 14-year follow-up and thereafter | 1 48% 2. 42% 3. 10% |
| Oh , 2021 | 1132 | Latent Profile analysis (used in previous study) | AIC, BIC, entropy | South Korea | Pregnancy | 4; prenatal, 6 months, 12 months, 24 months | Keshler K-6 | 3 | Group 1= none Group 2=Mild Group 3=Moderate | No symptoms group had Keshler scores less than 10 throughout the timepoints.Mild depression group had scores hovering around 12 throughout timepoints, peaking at 2 years. Moderate depression had highest scores throughout timepoints, peaking at slightly over 18 on the 1 year timepoint | Group 1=478 Group 2= 534 Group 3= 120 |
| Chae , 2020 | 1790 | Growth mixture modeling | AIC, BIC, full information maximum likelihood (FIML), LMR, SA-BIC | Chile | Postpartum | 4; when children were 1, 5, 10, and 14.6 years of age | CES-D | 4 | Class 1: infrequent Class 2: Increasing at adolescence Class 3: Decreasing at adolescence 4. Chronic Severe | Class 1 mothers consistently had infrequent depressive symptoms. Class 2 mothers had borderline-high depressive symptoms at child ages 1, 5.5 and 10, with scores increasing substantially from child age 10 years to adolescence. Class 3 mothers had steadily increasing depressive symptoms from child age 1 to 10, with symptoms decreasing dramatically from child age 10 years to adolescence. Class 4 mothers had consistently very high depressive symptoms throughout their child’s development | Class 1= 698, Class 2= 251, Class 3= 180 Class 4= 144 |
| Chow , 2019 | 3307 | Growth mixture model | , BIC, entopy, bootstrap likelihood ratio, full information maximum likelihood estimation | Canada | Pregnancy | 6; at recruitment during the second or third trimester (wave 1), at 36 weeks of gestation (wave 2), and at 6, 12, 18, and 24 months (waves 3–6) in the postnatal period | CES-D | 5 | 1. Persistent 2. Antepartum 3. Postpartum 4. Never (moderately low) 5. never (low) | 1. persistently high depressive symptoms 2. depressive symptoms particularly high in the antepartum period 3. high depressive symptoms present only after delivery, 4. moderately low depressive symptoms, always less than cut-off score 5. lowest levels of depressive symptoms, always less than cut-off score | 1. 2.3% 2.5.4% 3. 6.7% 4. 27.3% 5. 58.4% |
| Puosi , 2022 | 3808 | Latent growth mixture modelling | AIC, BIC, entropy | Finland | Pregnancy | 4; gestation weeks 14, 24, 34, and at child age 24 months | EPDS | 5 | 1.Consistently low 2.lowand increasing 3. Moderate and stable 4.consistently high 5. high and decreasing |  | 1.Consistently low=879 2.low and increasing=25 3. Moderate and stable=291 4.consistently high=43 5. high and decreasing=48 |
| van der Waerden , 2017 | 1899 | Semiparametric mixture model | AIC, BIC, entropy | France | Pregnancy | 5; 4 months, 8 months, 12 months, 3 years, 5 years | EPDS and CES-D | 5 | 1. No symptoms 2. Symptoms in pregnancy only 3. Symptoms during the child's preschool period 4. Persistent-intrmediate level symptoms 5. Persistent high level symptoms |  | 1. 645 2.38 3. 50 4.257 5.49 |
| van der Waerden , 2015 | 1899 | Growth trajectory models | BIC | France | Pregnancy | 5; 4 months, 8 months, 12 months, 3 years, 5 years | EPDS and CES-D | 5 | 1. no symptoms 2. persistent intermediate-level depressive symptoms 3. persistent high depressive symptoms 4. high symptoms in pregnancy only 5. high symptoms in the child’s preschool period only |  | 1. 736 2. 297 3. 54 4.42 5.54 |
| van der Waerden , 2015 | 1899 | Semi parametric group based modelling | BIC | France | Pregnancy | 5; 4 months, 8 months, 12 months, 3 years, 5 years | EPDS and CES-D | 5 | 1. no symptoms 2.pregnancy only 3. preschool only 4. intermediate 5. persistent high | 1. mothers had no symptoms 2. had high symptoms in pregnancy only 3. had high symptoms in the child’s preschool period only 4. had persistent intermediate-level symptoms 5. mothers had persistent high-level symptoms | 1. 1087 2. 85 3.89 4.456 5.90 |
| Betts , 2014 | 7223 | Latent class growth analysis | BIC, LMRT, bootstrap likelihood ratio test (BLRT) | Australia | Pregnancy | 4; prenatal, 3-5 days after birth, 6 months, 5 years | DSSI and Redeer Stress Inventory | 7 | (1) depressive, anxious, and stress symptoms during pregnancy (i.e., the prenatal group), (2) during birth, (3) at 6 months, and (4) at 5 years; , (5) ongoing depressive, anxious, and stress over the entire period, (6) ongoing stress symptoms (only) over the entire period, and (7) a normative group | (1) depressive, anxious, and stress symptoms during pregnancy (i.e., the prenatal group), (2) during birth, (3) at 6 months, and (4) at 5 years (5) ongoing depressive, anxious, and stress over the entire period, (6) ongoing stress symptoms (only) over the entire period, and (7) a normative group (not shown) | 1= 5.8% 2= 1.8% 3= 5.1% 4=7.4% 5=5.4% 6=3.1% |
| Betts , 2015 | 7223 | Latent class growth analysis | BIC, boostrap likelihood ratio, full information maximum likelihood (FIML), BLRT, entropy | Australia | Pregnancy | 4; prenatal, 3-5 days after birth, 6 months, 5 years | DSSI and Redeer Stress Inventory | 7 | (1) depressive, anxious, and stress symptoms during pregnancy (i.e., the prenatal group), (2) during birth, (3) at 6 months, and (4) at 5 years; , (5) ongoing depressive, anxious, and stress over the entire period, (6) ongoing stress symptoms (only) over the entire period, and (7) a normative group |  | 1. 381 2. 76 3. 279 4. 429 5. 320 6.190 7. 5074 |
| Drozd , 2018 | 1374 | Latent Growth curve | AIC, BIC, sample-size adjusted BIC, Lo-Mendell-Rubin (LMR) test, LMR adjusted likelihood ratio test (LMR-LRT), and the bootstrapped likelihood ratio test (BLRT), entropy, | Norway | Pregnancy and postpartum | 4; 1.5, 4, 6, and 12 months postpartum | EPDS | 2 | 1. High risk 2. Low risk | Low risk= accounted for 90% of the sample had a low symptom level that declined with time. high risk= had persistent depressive scores across time, slightly below the cut-off for a minor depression. | Low risk= 1249 High risk=119 |
| Barboza , 2021 | 682 | Growth mixture modeling | RMSEA, CFI, SRMR, Chi squared, AIC, BIC, Entropy | USA | Pregnancy | Last trimester of pregnancy and 4, 6, 8, 12, 18, 24, 30, and 36 months postpartum | BDI-II and timepoints | 3 | 1. Low risk \| 2. Early risk \| . Late risk | Class 1 (88.8%) is labeled the low-risk class characterized by decreasing depressive symptoms over time and stable high levels of PSE; class 2 (6.3%) is the early-risk class characterized by initially high levels of depressive symptoms that decline over time and moderate levels of PSE; and class 3 (4.9%) is the late-risk class characterized by increasing depressive symptoms and decreasing PSE throughout the study period | 1= 557 \| 2= 89 \| 3= 34 |
| Fisher , 2019 | 507 | Generalized linear mixed effect models | BIC, entropy, | USA | Postpartum | 4; baseline (4–8 weeks), 3, 6, and 12 months postpartum | SCID and SIGH-ADS | 3 | 1. Gradual remission 2. Partial improvement 3. Chronic severe depression | gradual remission trajectory= The SIGH-ADS scores decreased between baseline and 3 months postpartum, and gradually reached remission at 12 months. Partial improvement= Women experienced a consistent linear decrease in symptoms from moderate depression to mild depression at 12 months, which demonstrates improved but clinically significant symptoms. Chronic severe depression trajectory experienced a worsening of symptoms at 3 months postpartum to severe depression which was sustained | 1. 262 2. 211 3.34 |
| Giallo , 2017 | 1507 | Latent class analysis | AIC, BIC, Lsquare. Entropy, Voung-Lo, Mendell-Rubin | Australia | Pregnancy | 6; pregnancy and 3-, 6-, 12- and 18-months, and 4 years postpartum | EPDS | 3 | 1. Minimal symptoms 2. Sub-clinical symptoms 3. Persistent high symptoms | Minimal symptoms= women reported no or minimal depressive symptoms from pregnancy to 4 years postpartum (EPDS scores 2–4), Subclinical= reported subclinical symptoms (EPDS scores 6–8), and Persistently high= reported persistently high symptoms (EPDS scores 12–15) | minimal=880 subclinical=492 persistently high=135 |
| Lee , 2014 | 844 | Latent growth modelling | BIC, entropy, % of smallest class | USA | Pregnancy | 5; 0-5, 6-11, 12-17, 18-23, and 24+ month postpartum | EPDS | 3 | 1. Stable low symptoms 2. Decreasing symptoms 3. Increasing symptoms | Stable low= This class was marked by consistently reporting the lowest EPDS score over time. The average depression scores at the five time points were 5.75 (SD = 3.42), 5.78 (SD = 3.10), 5.96 (SD = 3.44), 5.07 (SD = 3.25) and 5.49 (SD = 3.44). Decreasing= characterized by a decreasing score during the entire follow-up period. The average scores were 16.36 (SD = 3.01), 13.50 (SD = 3.34), 8.65 (SD = 3.21), 8.03 (SD = 4.00), and 6.13 (SD = 3.72), which may reflect gradual resolution of depressive symptoms. Increasing= characterized by a moderate increase in score over time. The average scores were 11.42 (SD = 3.91), 13.67 (SD = 3.36), 14.00 (SD = 4.47), 14.96 (SD = 2.37) and 14.93 (SD =3.26), suggesting that women in this group may be experiencing a clinical level of PPD. | 1. 696 2.62 3.86 |
| Barker , 2013 | 12152 | Longitudinal latent class analysis | BIC, the Lo–Mendell–Rubin likelihood ratio test (LMR-LRT) and entropy | UK | Pregnancy | 5;(at 32 weeks prenatal, and 8 weeks, 8 months, 21 months and 33 months postnatal) | EPDS | 3 | 1. Chronic depressed 2. Medium symptoms 3. Low depressed |  | 1. 12151 2.4374 3.6561 |
| Giallo , 2015 | 1507 | Latent class analysis | Likelihood ratio statistic (L2), BIC, AIC, entropy, The Vuong-Lo-Mendall-Rubin likelihood, posterior probabilities | Australia | Pregnancy | 4; 10–24 weeks’ gestation, 3, 6, 12, and 18 months and 4 years postpartum | EPDS | 3 | Class1: Minimal depressive symptoms Class 2:Subclinical depressive symptoms 3. Increasing and persistently high depressive symptoms | ‘minimal depressive symptoms’ from pregnancy to 4 years postpartum (EPDS scores ranging 2–3). Class 2: ‘subclinical depressive symptoms’ over time (EPDS scores ranging 6–8). Class 3: ‘increasing and persistently high depressive symptoms’ over time (EPDS scores ranging 10–14). It is worth noting that, on average, women assigned to this third class had scores below the clinical cut-off of 13 on the EPDS during pregnancy and at 3 months postpartum, but these increased and remained high from 6 months to 4 years postpartum | Class 1= 662 Class 2= 328 Class 3= 95 |
| Pellowski , 2019 | 1225 | Group based trajectory models | BIC, posterior probabilities | South Africa | Pregnancy | 5; the second trimester of pregnancy, and 10 weeks, 6 months, 12 months, and 18 months postpartum | EPDS | 5 | group 1 (mild during pregnancy, slight decrease postpartum) group 2 (Minimal during pregnancy, increasing postpartum), group 3 (Unstable,peak at 12 months postpartum), group 4 (Moderate during pregnancy, minimal postpartum), group 5 (Severe during pregnancy and postpartum) | Group 1 = mild levels of depressive symptoms during pregnancy that did not meet the EPDS threshold for probable depression. symptoms decrease slightly postpartum but were still elevated. Group 2= started with low levels of depressive symptoms during pregnancy, which increased postpartum and were above the threshold for probable depression by 12 and 18 months postpartum. Group 3= participants had mild, but below threshold, levels of symptoms during pregnancy which decreased early postpartum (10 weeks). Later postpartum these symptoms increase peaking at 12 months postpartum with very severe levels of symptoms, and then dissipated to little to no depressive symptoms by 18 months postpartum. Group 4= characterized by moderate levels of depressive symptoms during pregnancy that decrease substantially postpartum. Group 5= persistent high levels of depressive symptoms that meet EPDS threshold for probable depression and symptoms persisted over time | 1. 82.9% 2.3.7% 3.6.6% 4.3.5% 5.3.1% |
| Hammerton, 2015 | 13617 | Latent Class Growth Modelling | SSABIC, Entropy, LMR-LRT | UK | Pregnancy | 10; 18 weeks gestation, 32 weeks gestation, 8 weeks postnatal, 8 months postnatal, 1 year 9 months, 2 years 9 months, 5 years 1 month, 6 years 1 month, 8 years 1 month and 11 years 2 months | EPDS | 5 | 1. Minimal 2. Mild 3. Increasing 4. Sub-threshold 5. Chronic | Minimal class: very low levels of depression symptoms over time Mild class: Nearly 18% belonged to a class with sub-threshold symptoms over time, with symptom levels that were consistently just below the clinical cut-off on the EPDS and decreased very slightly over time chronic severe class: high stable symptoms that were consistently above the clinical cut-off of 13 on the EPDS. Increasing class: increasing symptoms over time, with symptom levels that rose to the clinical cut-off by the last time point. Mild class: Just over 30% of the sample belonged to a class with stable mild symptoms over time | Minimal= 4177 Mild= 3384 Increasing= 583 Sub-threshold= 1863 Chronic= 552 |
